# Supplementary material for: 7 Tesla fMRI characterisation of the cortical-depth-dependent BOLD response in early human development
Source: bioRxiv. 2025 Aug 18:2025.08.18.670552. Preprint. [Version 1] doi: 10.1101/2025.08.18.670552 (PMC12393307; doi:10.1101/2025.08.18.670552)
Supplement: Supplement 1 [file NIHPP2025.08.18.670552v1-supplement-1.pdf]

bioRxiv preprint doi: https://doi.org/10.1101/2025.08.18.670552; this version posted August 19, 2025. The copyright holder for this preprint (which was not certified by peer review) is the author/funder, who has granted bioRxiv a license to display the preprint in perpetuity. It is made available under aCC-BY 4.0 International license.

Extended Data

Table S1. Kruskal-Wallis test to identify age-related differences in the ratio of maximum BOLD signal change across cortical depths in each trial between groups.

Ratio	Kruskal-Wallis test	
	H-statistic, *H*(3)	*p*-value
Superficial:Middle	35.12	<0.001
Middle:Deep	7.06	0.070
Superficial:Deep	35.96	<0.001

Table S2. Post-hoc Dunn test (with Bonferroni correction for multiple comparison) to identify age-related differences in ratio of maximum BOLD signal change between superficial and middle depths.

**Superficial:Middle (*p*-values)**				
	**Preterm**	**Early-term**	**Late-term**	**Adult**
**Preterm**	1.000			
**Early-term**	1.000	1.000		
**Late-term**	1.000	0.091	1.000	
**Adult**	<0.001	<0.001	0.024	1.000

bioRxiv preprint doi: https://doi.org/10.1101/2025.08.18.670552; this version posted August 19, 2025. The copyright holder for this preprint (which was not certified by peer review) is the author/funder, who has granted bioRxiv a license to display the preprint in perpetuity. It is made available under aCC-BY 4.0 International license.

Table S3. Post-hoc Dunn test (with Bonferroni correction for multiple comparison) to identify age-related differences in ratio of maximum BOLD signal change between superficial and deep depths.

Superficial:Deep (*p*-values)

	**Preterm**	**Early-term**	**Late-term**	**Adult**
**Preterm**	1.000			
**Early-term**	1.000	1.000		
**Late-term**	0.174	0.041	1.000	
**Adult**	<0.001	<0.001	0.104	1.000
